# Free vitamin D correlate better with bone mineral density and thoracolumbar junction osteoporotic vertebral fractures than serum vitamin D

**DOI:** 10.1186/s12891-020-3179-7

**Published:** 2020-03-12

**Authors:** Kishor chhantyal, Lei He, Jian Mo, Mingyu Yin, Tianwei He, Yuyong Chen, Yang Yang, Liangming Zhang, Limin Rong

**Affiliations:** 1 Department of Spine Surgery, the Third Affiliated Hospital, Sun Yat-Sen University, No.600 Tianhe Road, Guangzhou, 510630 Tianhe District China; 2 Guangdong Provincial Center for Quality Control of Minimally Invasive Spine Surgery, No. 600 Tianhe Road, Guangzhou, 510630 China; 3 Guangdong Provincial Center for Engineering and Technology Research of Minimally Invasive Spine Surgery, No. 600 Tianhe Road, Guangzhou, 510630 China; 4 Department of Rehabilitation Medicine, the Third Affiliated Hospital, Sun Yat-Sen University, No. 600 Tianhe Road, Guangzhou, 510630 China

**Keywords:** Total vitamin D, Free vitamin D, Bone mineral density, Osteoporosis, Thoracolumbar junction vertebral fractures

## Abstract

**Background:**

Vitamin D deficiency has long been studied as a risk factor for osteoporosis. However, the association between serum vitamin D status, bone mineral density (BMD) and the incidence of vertebral fractures (OVFs) remain controversial. It is believed that free portion of the circulating vitamin D carries the metabolic activities of vitamin D. Therefore, the aim of the present study is to analyse if free vitamin D correlates with BMD and osteoporotic fragile vertebral fractures in the elderly population.

**Methods:**

A total of 90 consecutive patients, including 81 female and 9 male patients, aged > 48 years, were included in this cross sectional study between March and July of 2018. Total vitamin D (total 25(OH)D), free vitamin D (free 25(OH)D), calcium and phosphorus were measured. BMD was measured using dual energy X-ray absorptiometry (DEXA) and osteoporotic vertebral fracture was assessed using plain radiograph. Multiple linear regression was performed to find out the association between total vitamin D, free vitamin D and BMD at various sites. To evaluate the association with osteoporotic vertebral multivariate logistic regression model was used.

**Results:**

The mean total vitamin D and free vitamin D were 25.1 ± 10.2 and 6.1 ± 1.7 respectively. Free vitamin D had a linear correlation with total vitamin D (R^2^ = 0.69). While free vitamin D had a positive correlation with lumbar BMD roles (*p* < 0.05), total vitamin D didn’t have any association with BMD at any site. Of the total patients, 62 patients (68.9%) had thoracolumbar junction OVFs. Free vitamin D level correlated with the prevalence of OVFs as well as lumbar osteoporosis (*p* < 0.05). However, there was no statistical correlation between serum vitamin D status and the OVFs.

**Conclusions:**

Free vitamin D was significantly related to the occurrence of thoracolumbar junction OVFs and lumbar BMD, which assumed to be a positive predictor for fracture and osteoporosis prevention. However, total serum vitamin D levels did not have any association with BMD at different sites as well as fragile vertebral fracture.

**Trial registration:**

The study is registered at clinicaltrials.gov NCT03605173.

## Background

Osteoporosis is an inevitable chronic condition that results in the reduction of the bone mineral density and fragility of the bone. It is the predisposing factor for millions of fractures worldwide every year. Depending on the severity and location, fractures can lead to many complications, such as disability, increased dependency, poor quality of life and increased burden of healthcare costs [1]. Some well-known risk factors for osteoporosis include age, sex, ethnicity, family history, renal insufficiency and alcohol dependency, smoking and lack of physical activity [2]. Over the years, various nutritional factors including vitamin D has been extensively studied to find out the possible role in osteoporosis [3].

Vitamin D is a fat-soluble pro-hormone obtained through skin exposure to sunlight and from dietary intake. This vitamin is bound to vitamin D-binding protein (DBP) in circulation and undergoes hydroxylation first in the liver and then in the kidney to form 25-hydroxy vitamin D (25(OH)D) and active hormone 1,25-dihydroxyvitamin D (1,25(OH)_2_D) respectively. Vitamin D is responsible for intestinal calcium absorption, bone calcium resorption and renal calcium reabsorption to maintain calcium homeostasis and promote skeletal mineralization [4]. Its deficiency often results in skeletal pathologies such as rickets and osteomalacia [5]. Currently, in clinical practice, measuring serum 25(OH) D concentration is considered to be the best estimation of Vitamin D status [6].

Observational studies have suggested associations between deficiency or insufficiency of vitamin D and secondary hyperparathyroidism, elevation of bone turnover markers and bone loss [7]. The degradation of the bone quality and low BMD levels are robust predictors of osteoporotic fracture. While several studies have found a positive association between low serum vitamin D and low BMD at various sites and different populations [8–10], other studies did not find any significant relationship between these two parameters [11, 12]. Likewise, few studies have focused on the association between vitamin D and osteoporotic vertebral fractures (OVFs), which frequently occur at the thoracolumbar junction [12]. The contradicting results of association of serum 25(OH) D with BMD and fragile vertebral fracture have led to the study of possible role of free vitamin D, which is the portion of vitamin D unbound to protein carrier in circulation, with these parameters. Therefore, the aim of the present study is to analyze if free vitamin D correlates with BMD and osteoporotic fragile vertebral fractures in the elderly population. It was hypothesized that free vitamin D but not the total serum vitamin D would be associated with factors mentioned above.

## Methods

### Subjects

Between March and July of 2018, a total of 283 patients with osteoporosis or OVFs admitted in the Department of Spine Surgery of the Third Affiliated Hospital of Sun Yat-sen University were eligible for the study. Of these eligible patients, a total of 90 patients were enrolled in this cross sectional study. Patients were included if they were male or post-menopausal female aged > 48 years. The exclusion criteria included 1) age < 48 years; 2) patients under vitamin D and calcium supplement in the recent 6 months; 3) patients with suspected hyperparathyroidism; 4) patients under steroid; 5) patients with chronic diseases such as liver cirrhosis, renal failure, hypertension, and diabetes. Those with malignancy were also excluded from the study. The study was approved by the institutional review board of the Third Affiliated Hospital of Sun Yat-sen University. Informed consent was obtained from all the participants.

### Participant characteristics

The demographic information was collected, including age, smoking history, alcohol intake, height, weight, and body mass index (BMI). Fasting venous blood was collected during March through July of 2018. These blood samples were drawn from patients in the morning on the day after admission. The samples were then centrifuged and the resulting serum was stored at − 80 °C. History of vitamin D and calcium supplement was noted during the admission procedures. Height and weight were measured with light clothing and no shoes.

### Total vitamin D and bone parameters

Total 25(OH) D measurements were analyzed by ELISA method by IDS EIA kit (IDS, Boldon, UK). Serum calcium and phosphorus concentration were examined by HITACHI 7060 Automatic Biochemical Analyzer (Hitachi Ltd., Japan). The lab technician was blinded to study outcomes.

### BMD

Dual-energy x-ray absorptiometry (by Hologic Discovery DEX A system (Hologic, Bedford, MA) scanning was used to assess hip, femoral neck and lumbar spine (L1–4) areal BMD g/cm2). As per the World Health Organization (WHO) classification system, osteoporosis is defined as T-score ≤ − 2.5, osteopenia as − 2.5 < T-score < − 1, and normal as T-score ≥ − 1. All scans were accomplished by a senior musculoskeletal radiologist.

### Free vitamin D

Free 25(OH) D concentrations were determined by immunoassay (Future Diagnostics B.V., Wijchen, The Netherlands, http://www.future-diagnostics.nl/). In this assay, the microplate is coated with an anti-vitamin D antibody. During a first incubation, the Free 25(OH) D is captured by the antibody. Following a wash as per essay protocol, during a second incubation, a biotin-labeled 25(OH) D analog and non-occupied antibody binding sites are allowed to react with each other. The bound enzyme is then quantitated using a colorimetric reaction, following washing and incubation with a streptavidin peroxidase conjugate. The intensity of the signal is inversely proportional to the level of free 25(OH) D in the sample. The assay was calibrated against a symmetric dialysis method. The assay was calibrated over the range of 0.1 to 35 pg/mL with a limit of detection of 2.8 pg/mL; inter- and intra-assay CVs were < 10%.

### Osteoporotic vertebral fracture

The anteroposterior and lateral thoracolumbar spine or chest X-ray films were used to assess the vertebral fractures. The vertebrae could be visualized by adjusting the bone window parameters on picture archiving and communication system (PACS). The thoracolumbar OVFs were defined by using the Genant semiquantitative (SQ) approach. According to Genant SQ scale [13], OVFs were graded as follows: a) Grade 1, a reduction in vertebral height of 20–25%; b) Grade 2, a reduction of 26–40%; c) Grade 3, a reduction of over 40%. We classified Grade 1 and Grade 2 as mild fracture, Grade 3 as severe fracture. Each X-ray image was evaluated by two experienced spine surgeon (> 10 years of experience) independently to assess whether it contained a vertebral fracture. Preliminary experiments were carried out to standardize the image diagnosis for the sake of consistency of final results.

### Statistical analysis

Demographic and baseline characteristics are presented as mean ± S. D. The characteristics of BMD and fracture parameters with total 25(OH) D and free 25(OH) D were analyzed using student’s t-test as appropriate. Relationship between total and free 25(OH) D concentrations was tested by plotting a scattered plot graph as well as Pearson’s correlation. To determine the relationship of bone health parameters with total 25(OH) D and free 25(OH) D, Pearson’s correlation coefficient was determined. Multiple linear regression with age, gender, and BMI as a covariate was performed with bone health parameter as a dependable variable. Multivariate logistic regression model was used to evaluate the association between total and free 25(OH) D with osteoporosis and OVFs after adjusting age, gender and BMI. The receiver operating characteristic (ROC) curve was performed to determine the diagnostic value of free 25(OH) D in distinguishing the osteoporosis at different sites and also the OVFs. A *p*-value < 0.05 was considered statistically significant, and all tests were two-sided. SPSS 21.0 software was used for statistical analysis (SPSS, Chicago, USA) and R version 3.6.2 software was used for comparison of areas under the ROC curves (The R Foundation, USA).

## Results

The baseline characteristics of the total 90 participants, which included 81 female and 9 male participants were listed in Table 1. The mean total vitamin D, free vitamin D, calcium and phosphorus were 25.1 ± 10.2 ng/ml, 6.1 ± 1.7 pg/ml, 9.3 ± 0.5 mg/dL and 3.5 ± 0.5 mg/dL respectively. Of the total recruited participants, 83 (92.2%) were osteoporotic.

**Table 1:** Characteristics of participants

Characteristics	Mean	SD
Age (years)	68.5	10.0
BMI	22.6	3.6
Height (m)	1.55	0.79
Weight (kg)	54.4	10.3
Systolic pressure (mmHg)	127.2	16.7
Diastolic pressure (mmHg)	78.4	8.7
Total 25(OH) D (ng/ml)	25.1	10.2
Free 25(OH) D (pg/ml)	6.1	1.7
Calcium (mg/dL)	9.3	0.5
Phosphorus (mg/dL)	3.5	0.5
Lumbar T-score	−3.8	1.1
Femur neck T-score	−2.8	1.2
Hip T-score	−2.6	1.2
Lumbar BMD	0.6	0.1
Femur neck BMD	0.5	0.1
Hip BMD	0.6	0.2
Gender (N, %)		
Male	9	10.0
Female	81	90.0
Fracture (N, %)		
No	28	31.1
Yes	62	68.9
Fracture Grade (N, %)		
1	7	7.8
2	28	31.1
3	27	30.0
Fracture Times (N, %)		
Single Fracture	41	45.6
Re-Fracture	21	23.3

### Relationship between Total and free vitamin D

**Fig. 1:**
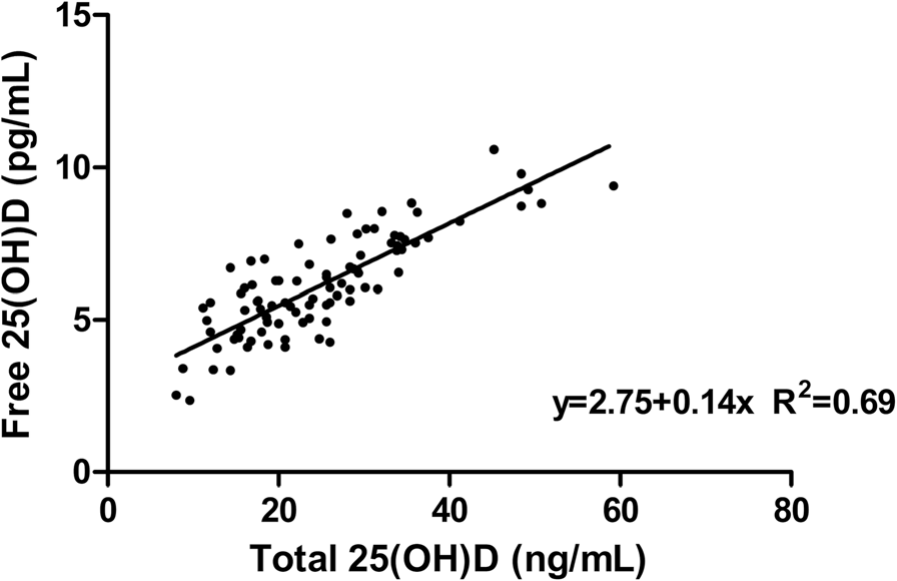
Total 25(OH) D concentrations are plotted on the x-axis, and directly measured free 25(OH) D levels are plotted on the y-axis. Directly measured free concentrations were related to total 25(OH) D concentrations (R^2^ = 0.69, *p* < 0.01). The line represents the best linear fit

When plotted a scattered plot graph (Fig. 1), free vitamin D had a linear correlation with total vitamin D (r = 0.83, 95%CI: 0.76–0.88). The Pearson’s correlation showed that the free vitamin D had a significant positive correlation with the total vitamin D (*p* < 0.01).

### Associations with BMD

To explore the associations between vitamin D parameters and BMD at all sites, we conducted a multiple linear regression analysis. Greater free vitamin D was independently associated with greater lumbar BMD. However, it did not have any association with BMD at hip and femur neck. Total vitamin D did not have significant association with BMD at all sites. (Table 2).

**Table 2:** Associations between Vitamin D and BMD at Lumbar, Femur neck, and Hip

Dependent variables:	Total 25(OH)D		Free 25(OH)D	
	β(95% CI)^a^	*p* value	β(95% CI) ^a^	*p* value
Lumbar BMD	0.002 (0.000, 0.004)	0.093	0.016 (0.002, 0.030)	0.030^*^
Femur neck BMD	0.002 (0.000, 0.004)	0.081	0.008 (−0.007, 0.023)	0.290
Hip BMD	0.001(−0.002, 0.004)	0.433	0.002 (−0.015, 0.019)	0.798
Lumbar T score	0.002(− 0.028, 0.032)	0.912	0.115 (− 0.070, 0.299)	0.219
Femur neck T score	0.014(−0.011, 0.039)	0.276	0.118 (−0.038, 0.274)	0.135
Hip T score	0.013(−0.009, 0.035)	0.254	0.024 (−0.112, 0.160)	0.727

*CI* Confidence interval

^a^ multiple linear regression model: adjusted for age, gender, BMI

^*^means statistically significant values (*p* < 0.05)

### Associations with osteoporosis

**Fig. 2:**
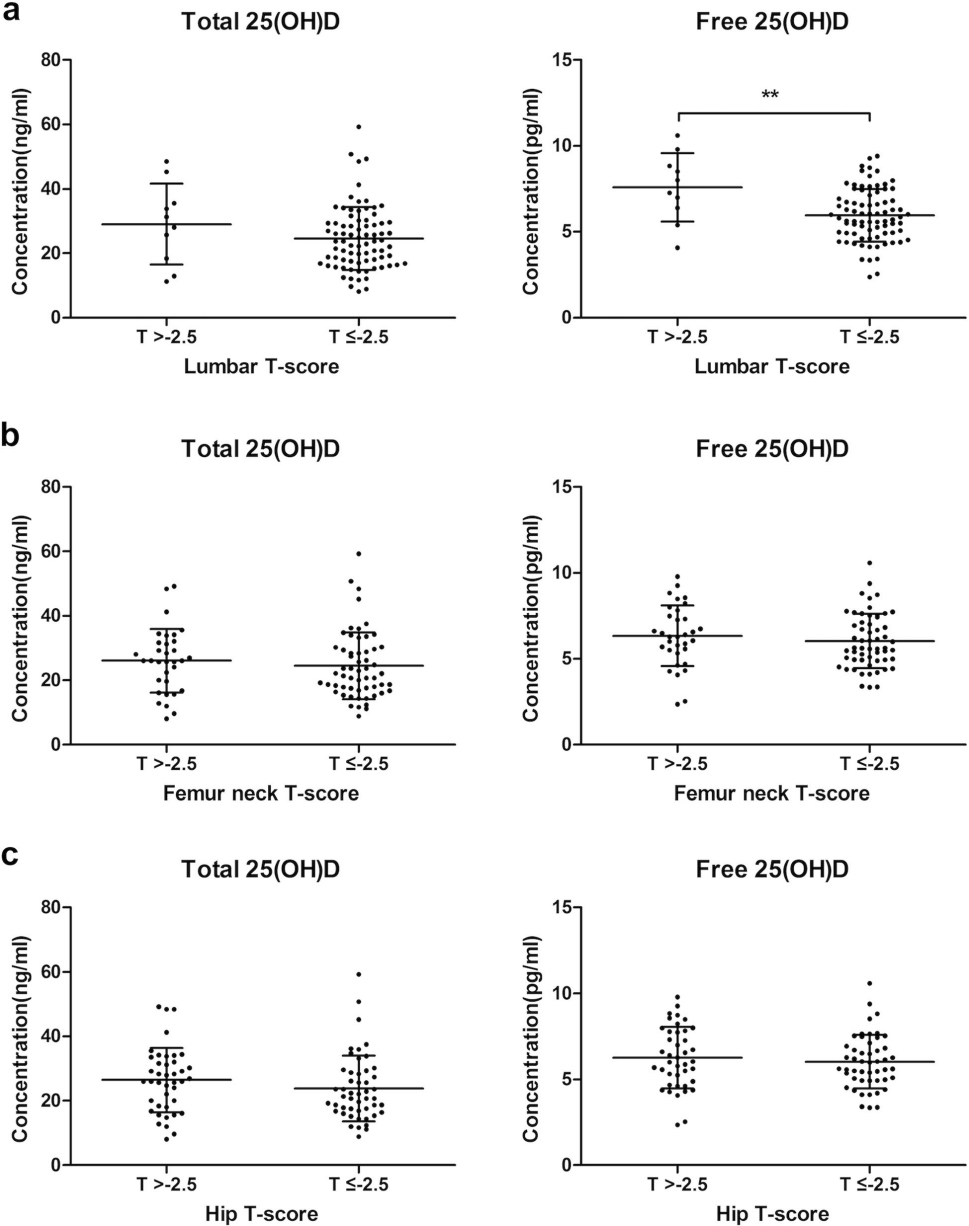
Scatter plots of total and free 25(OH) D concentrations plotted against T-SCORE at different sites; (**a**) Lumbar (**b**) Femur neck (**c**) Hip. The concentration of free 25(OH) D was considerably lower in lumbar osteoporotic group compared to non-osteoporotic group. The black bars indicate mean and standard deviation. **p < 0.01

We divided the T-scores of all sites into two different groups; non-osteoporotic (T-score > − 2.5) and osteoporotic (T-score ≤ − 2.5). As seen on the scatter plots, decreasing trend of free vitamin D levels was observed between lumbar non-osteoporotic group (7.6 ± 2.0 pg/ml) and lumbar osteoporotic group (6.0 ± 1.5 pg/ml) (*p* < 0.01) (Fig. 2). To further explore the association between vitamin D and osteoporosis, the multivariate logistic model adjusting for age, gender and BMI was conducted. Free vitamin D level correlated with the prevalence of lumbar osteoporosis (Table 3). However, there was no statistically significant correlation between total vitamin D and osteoporosis. In addition, the ROC curve showed that the free 25(OH) D was more powerful predictor for lumbar osteoporotic than total vitamin D (area under the curve: 0.738 > 0.609, *p* < 0.05) (Fig. 4a).

**Table 3:** Associations between Vitamin D and Osteoporosis

Dependent variables:	Total 25(OH)D		Free 25(OH)D	
	Adjusted Odd ratio(95% CI)^a^	*p* value	Adjusted Odd ratio(95% CI)^a^	*p* value
Lumbar osteoporotic	0.963(0.906, 1.023)	0.218	0.550(0.349, 0.867)	0.010^*^
Femur neck osteoporotic	0.986 (0.940, 1.034)	0.554	0.915 (0.684, 1.223)	0.547
HIP osteoporotic	0.971 (0.925, 1.021)	0.251	0.942 (0.706, 1.255)	0.682
Any site osteoporotic	0.948 (0.883, 1.017)	0.138	0.578 (0.346, 0.966)	0.036^*^

*CI* Confidence interval

^a^ multivariate logistic model: adjusted for age, gender, BMI

^*^means statistically significant values (*p* < 0.05)

### Associations with OVF

**Fig. 3:**
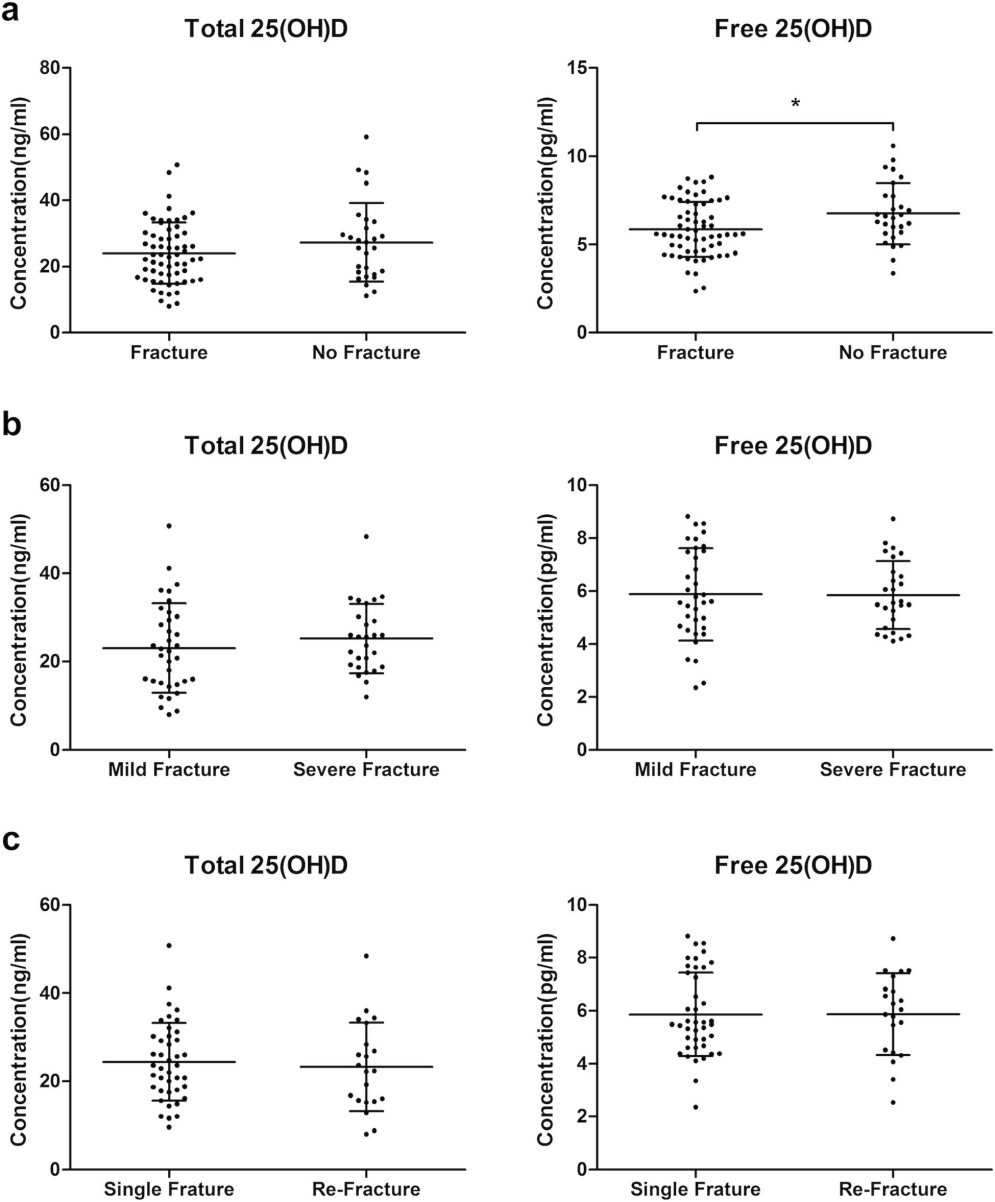
Scatter plots of total and free 25(OH) D concentrations plotted against different fracture parameters; (**a**) presence of fracture (**b**) fracture grade **c**) occurrence of fracture. Free vitamin D concentration was considerably lower in patients with OVFs than in patients without OVFs. The black bars indicate mean and standard deviation. *p < 0.05. OVFs: osteoporotic vertebral fractures

**Fig. 4:**
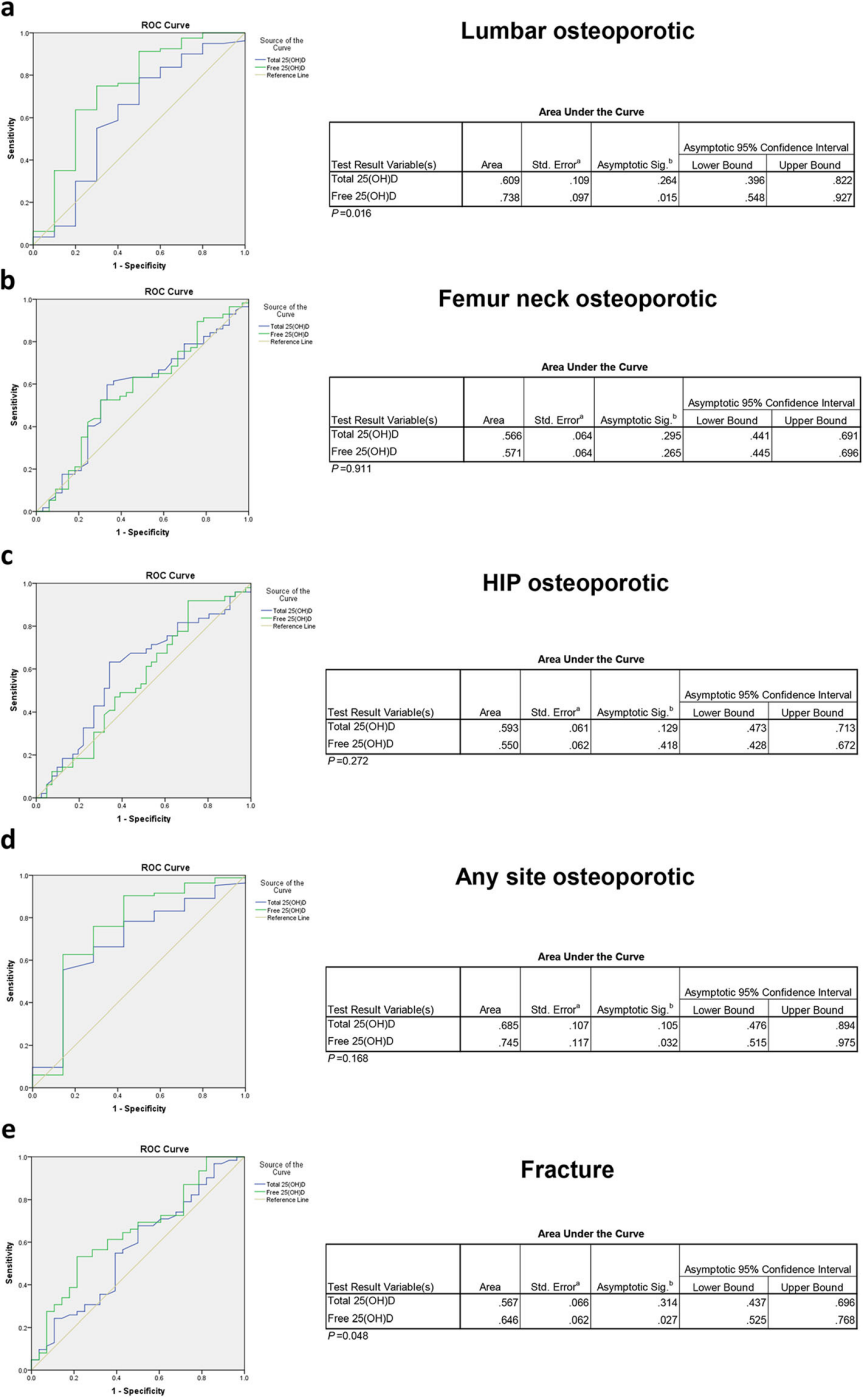
The receiver operating characteristic (ROC) curve was performed to determine the diagnostic value of free 25(OH) D in distinguishing the osteoporosis at different sites and also the OVF

The thoracolumbar OVFs were assessed on the thoracolumbar spine or chest X-ray images using the PACS system. In our study, 35 patients (38.9%) had mild OVFs (7 in Grade 1 and 28 in Grade 2), and 27 patients (30.0%) had severe OVFs (Grade 3). Regarding the fracture times, while 41 patients (45.6%) had OVFs for the first time, 21 patients (23.3%) had vertebral re-fracture (Table 1). Free vitamin D concentration was found to be lower in 62 patients with OVFs (5.9 ± 1.6 pg/ml) than patients without OVFs (6.8 ± 1.7 pg/ml) (*p* < 0.05) (Fig. 3). Multivariate logistic regression for vitamin D and OVF generated similar results showing that there was a significant correlation between free vitamin D and OVFs. Free vitamin D was assumed to be a positive predictor for fracture prevention. However, the occurrence of OVFs did not have any association with total vitamin D level. Neither total nor free vitamin D had significant relationship with vertebral fracture grade and re-fracture (Table 4). The ROC curve showed that the free 25(OH) D was more powerful predictor for lumbar OVF than total vitamin D (area under the curve: 0.646 > 0.567, *p* < 0.05) (Fig. 4e). The average ICC value for the OVF readings was 0.87 for interobserver reliability, 0.86 and 0.89 indicating intraobserver reliability for the two viewers, respectively.

**Table 4:** Associations between Vitamin D and Osteoporotic Vertebral Fracture

Dependent variables:	Total 25(OH)D		Free 25(OH)D	
	Adjusted Odd ratio(95% CI)^a^	*p* value	Adjusted Odd ratio(95% CI)^a^	*p* value
Fracture	0.967 (0.924, 1.012)	0.152	0.696 (0.513, 0.943)	0.019^*^
Fracture Grade	1.031 (0.972, 1.093)	0.317	1.003 (0.713, 1.413)	0.985
Re-Fracture	0.998 (0.938, 1.062)	0.949	1.060 (0.735, 1.529)	0.755

CI confidence interval

^a^ multivariate logistic model: adjusted for age, gender, BMI

^*^means statistically significant values (*p* < 0.05)

## Discussion

Free vitamin D is the portion of vitamin D in circulation which is unbound to protein. Around 85–90% of the total vitamin D in circulation is bound to its specific binding protein (DBP) and weakly to plasma albumin (10–15%), leaving a small fraction (< 1%) as its “free form” [14–16]. The “free-hormone hypothesis” states that the biologic activity of hormones is carried out by its free fractions, which is unbound to protein carrier [17]. Free hormone concentration is routinely evaluated to evaluate the in vivo activity of sex and thyroid hormones and, and an increasingly large cellular, physiological and biochemical data strongly support the free hormone hypothesis. It has been suggested that there is an impaired delivery of 25(OH) D to vitamin D-activating 1-alpha-hydroxylase in target cells when bound to the protein carrier, and thus it is hypothesized that the functional status of the vitamin D is best reflected by its free proportion. Thus, measuring total serum 25(OH) D to assess the vitamin D status is questionable [18]. The principal objective of this study was to analyze whether free vitamin D would correlate better with BMD than total vitamin D; and secondly, to examine if free vitamin D was a better determinant of osteoporotic fragile vertebral fracture.

Lumbar BMD had a significant positive correlation with the free vitamin D, whereas no significant associations were seen for total vitamin D. However, neither free nor total vitamin D had a significant correlation with BMD at hip and femur neck. Our results are in agreement with those reported by a few other studies. Powe et al., who analyzed 49 male and female students between 18 and 31 years old, found that free and bio-available 25(OH) D had a significant correlation with BMD, and this was not seen with total 25(OH)D [19]. Similarly, Allison RJ et al. in his study of racially diverse athletic population found that bioavailable vitamin D had association with lumbar, neck and hip BMD, but no significant correlation between BMD and total vitamin D was observed [20]. Another study of postmenopausal women concluded that free and bioavailable vitamin D associated BMD, which was not seen between total vitamin D and BMD [21]. Therefore, free vitamin D could be a better measure than total vitamin D in relation to BMD.

The major difference between many similar studies and this study is that we measured the free vitamin D concentration directly using the immunoassay, which is a more accurate measurement of the free vitamin D level. The aforementioned studies had calculated the free vitamin D levels by using the formula rather than direct measurement [22]. It has been shown that there is a concordance between directly measured vs. calculated free vitamin D levels as well as has a dependent bias of concentration suggesting that the direct measure of free vitamin D concentrations could be a better option than the calculated one [23]. In another study, directly measured free vitamin D concentrations were related to parathyroid hormone (PTH) and calcium, but calculated estimates were not. The study found that the level of calculated free vitamin D varied significantly from the directly measured one. It also suggested that current formulas used to calculate free vitamin D may be inaccurate [24]. There are potential errors when calculating free vitamin D levels, and these errors could be avoided by using the direct measurement assay.

The relation between total vitamin D and fracture remains unclear and controversial. It is well-known fact that osteoporosis is one of the predisposing factors for fracture. In our study, only the thoracolumbar junctional vertebral fractures (T10-L2) were studied due to two obvious reasons. Firstly, the lower lumbar vertebrae could not be visualized in a chest x-ray, and secondly, in clinical practice, OVFs are more prevalent at the thoracolumbar junction. Vitamin D deficiency has been reported as a risk factor for osteoporotic fractures [25]. Recently, vitamin D insufficiency was concluded as a risk factor for fragile vertebral fractures in both men and women [26]. A study of community-dwelling postmenopausal women reported that repletion of vitamin D would reduce the risk of future fracture risk [27]. In contrast to other reports, in our study, we did not observe an association between total vitamin D and vertebral fracture.

Our results found a significant positive association between fragile vertebral fracture and free vitamin D. However, the reason and phenomenon for this remain unclear. Recently, it has been suggested that free vitamin D may be physiologically essential in human [28]. When the vitamin D was supplemented in deficient people, in the early course, the rise in free vitamin D level associated with the increase and decrease in 24,25(OH)_2_D and parathyroid hormone respectively. Perhaps, the positive association between lumbar BMD as well as the lumbar osteoporosis might explain this phenomenon to a certain extent. Even though most fractures are likely to occur in osteopenic population, epidemiological studies have suggested that elderly people with a fracture will have BMD in the osteoporotic range [29]. Hence, free vitamin D may be an independent risk factor for vertebral fractures. To our knowledge, there have been no previous reports which have focused on the association between free vitamin D and fragile vertebral fractures.

The significant association of free vitamin D with lumbar BMD and fragile vertebral fracture is the major strength of the present study. However, there are some limitations to this study. First, the sample size is relatively small; especially that of male participants. Moreover, all the patients included in the study are from southern China, and hence, the racial and geographical variation of free vitamin D could not be studied. We also did not have any data for vitamin D binding protein and albumin. However, these measures would not have an effect on directly measured free vitamin D levels compared to calculated value. Also, the cross-sectional design prevents any discussion about causal relationships. Furthermore, the study was completed among patients with OVF or osteoporosis so it is unclear if these findings would be applicable in the general population.

## Conclusions

Free vitamin D was significantly related to the occurrence of thoracolumbar junction OVFs and lumbar BMD, which assumed to be a positive predictor for fracture and osteoporosis prevention. However, total serum vitamin D levels did not have any association with BMD at different sites as well as fragile vertebral fracture.

## Data Availability

The datasets used and/or analyzed during the current study are available from the corresponding author on reasonable request.

## Abbreviations

1,25(OH)_2_: 1,25-dihydroxy vitamin D
25(OH)D: 25-hydroxy vitamin D
BMD: Bone mineral density
BMI: Body mass index
DBP: D-binding protein
OVF: Osteoporotic vertebral fracture
PACS: Picture archiving and communication system
PTH: Parathyroid hormone
ROC: Receiver operating characteristic
SQ: Semiquantitative
WHO: World Health Organization

## References

[1] Johnell O, Kanis JA (2006). An estimate of the worldwide prevalence and disability associated with osteoporotic fractures. Osteoporos Int.

[2] Anagnostis P, Karagiannis A, Kakafika AI, Tziomalos K, Athyros VG, Mikhailidis DP (2009). Atherosclerosis and osteoporosis: age-dependent degenerative processes or related entities?. Osteoporos Int.

[3] Tucker KL, Morita K, Qiao N, Hannan MT, Cupples LA, Kiel DP (2006). Colas, but not other carbonated beverages, are associated with low bone mineral density in older women: the Framingham osteoporosis study. Am J Clin Nutr.

[4] Lieben L, Carmeliet G (2013). Vitamin d signaling in osteocytes: effects on bone and mineral homeostasis. Bone..

[5] Hamilton B (2010). Vitamin d and human skeletal muscle. Scand J Med Sci Sports.

[6] Holick MF (1990). The use and interpretation of assays for vitamin d and its metabolites. J Nutr.

[7] Didriksen A, Grimnes G, Hutchinson MS, Kjaergaard M, Svartberg J, Joakimsen RM (2013). The serum 25-hydroxyvitamin d response to vitamin d supplementation is related to genetic factors, bmi, and baseline levels. Eur J Endocrinol.

[8] Choi SW, Kweon SS, Choi JS, Rhee JA, Lee YH, Nam HS (2016). The association between vitamin d and parathyroid hormone and bone mineral density: the dong-gu study. J Bone Miner Metab.

[9] Nguyen HT, von Schoultz B, Nguyen TV, Dzung DN, Duc PT, Thuy VT (2012). Vitamin d deficiency in northern Vietnam: prevalence, risk factors and associations with bone mineral density. Bone..

[10] Pourhashem Z, Bayani M, Noreddini H, Bijani A, Hosseini SR (2012). Prevalence of osteoporosis and its association with serum vitamin d level in older people in amirkola, north of Iran. Caspian J Intern Med.

[11] Zhen D, Liu L, Guan C, Zhao N, Tang X (2015). High prevalence of vitamin d deficiency among middle-aged and elderly individuals in northwestern China: its relationship to osteoporosis and lifestyle factors. Bone..

[12] Bruno AG, Burkhart K, Allaire B, Anderson DE, Bouxsein ML (2017). Spinal loading patterns from biomechanical modeling explain the high incidence of vertebral fractures in the thoracolumbar region. J Bone Miner Res.

[13] Genant HK, Wu CY, van Kuijk C, Nevitt MC (1993). Vertebral fracture assessment using a semiquantitative technique. J Bone Miner Res.

[14] Bikle DD, Gee E, Halloran B, Kowalski MA, Ryzen E, Haddad JG (1986). Assessment of the free fraction of 25-hydroxyvitamin d in serum and its regulation by albumin and the vitamin d-binding protein. J Clin Endocrinol Metab.

[15] Yousefzadeh P, Shapses SA, Wang X (2014). Vitamin d binding protein impact on 25-hydroxyvitamin d levels under different physiologic and pathologic conditions. Int J Endocrinol.

[16] Bouillon R, Van Assche FA, Van Baelen H, Heyns W, De Moor P (1981). Influence of the vitamin d-binding protein on the serum concentration of 1,25-dihydroxyvitamin d3. Significance of the free 1,25-dihydroxyvitamin d3 concentration. J Clin Invest.

[17] Mendel CM (1989). The free hormone hypothesis: a physiologically based mathematical model. Endocr Rev.

[18] Chun RF, Peercy BE, Orwoll ES, Nielson CM, Adams JS, Hewison M (2014). Vitamin d and dbp: the free hormone hypothesis revisited. J Steroid Biochem Mol Biol.

[19] Powe CE, Ricciardi C, Berg AH, Erdenesanaa D, Collerone G, Ankers E (2011). Vitamin d-binding protein modifies the vitamin d-bone mineral density relationship. J Bone Miner Res.

[20] Allison RJ, Farooq A, Cherif A, Hamilton B, Close GL, Wilson MG (2018). Why don't serum vitamin d concentrations associate with bmd by dxa? A case of being 'bound' to the wrong assay? Implications for vitamin d screening. Br J Sports Med.

[21] Johnsen MS, Grimnes G, Figenschau Y, Torjesen PA, Almas B, Jorde R (2014). Serum free and bio-available 25-hydroxyvitamin d correlate better with bone density than serum total 25-hydroxyvitamin d. Scand J Clin Lab Invest.

[22] Vermeulen A, Verdonck L, Kaufman JM (1999). A critical evaluation of simple methods for the estimation of free testosterone in serum. J Clin Endocrinol Metab.

[23] Peris P, Filella X, Monegal A, Guanabens N, Foj L, Bonet M (2017). Concordance between direct and indirect measurements of free 25-oh vitamin d. Clin Chim Acta.

[24] Schwartz JB, Lai J, Lizaola B, Kane L, Markova S, Weyland P (2014). A comparison of measured and calculated free 25(oh) vitamin d levels in clinical populations. J Clin Endocrinol Metab.

[25] Sakuma M, Endo N, Oinuma T (2007). Serum 25-ohd insufficiency as a risk factor for hip fracture. J Bone Miner Metab.

[26] Maier GS, Seeger JB, Horas K, Roth KE, Kurth AA, Maus U (2015). The prevalence of vitamin d deficiency in patients with vertebral fragility fractures. Bone Joint J.

[27] LeBoff MS, Kohlmeier L, Hurwitz S, Franklin J, Wright J, Glowacki J (1999). Occult vitamin d deficiency in postmenopausal us women with acute hip fracture. Jama..

[28] Shieh A, Ma C, Chun RF, Wittwer-Schegg J, Swinkels L, Huijs T (2018). Associations between change in total and free 25-hydroxyvitamin d with 24,25-dihydroxyvitamin d and parathyroid hormone. J Clin Endocrinol Metab.

[29] Trajanoska K, Morris JA, Oei L, Zheng HF, Evans DM, Kiel DP (2018). Assessment of the genetic and clinical determinants of fracture risk: genome wide association and mendelian randomisation study. Bmj..

